# Closure of Earth’s Global Seasonal Cycle of Energy Storage

**DOI:** 10.1007/s10712-023-09797-6

**Published:** 2023-07-18

**Authors:** Gregory C. Johnson, Felix W. Landerer, Norman G. Loeb, John M. Lyman, Michael Mayer, Abigail L. S. Swann, Jinlun Zhang

**Affiliations:** 1 NOAA/Pacific Marine Environmental Laboratory, Seattle, WA 98115 USA; 2 Jet Propulsion Laboratory, California Institute of Technology, Pasadena, CA 91109 USA; 3 NASA Langley Research Center, Hampton, VA 23681 USA; 4 CIMAR, University of Hawaii, Honolulu, HI 96822 USA; 5 Research Department, ECMWF, 53175 Bonn, Germany; 6 Department of Meteorology and Geophysics, University of Vienna, 1090 Vienna, Austria; 7 Departments of Atmospheric Sciences and Biology, University of Washington, Seattle, WA 98195 USA; 8 Applied Physics Laboratory, University of Washington, Seattle, WA 98105 USA

**Keywords:** Earth, Climate, Global energy, Seasonal cycle

## Abstract

The global seasonal cycle of energy in Earth’s climate system is quantified using observations and reanalyses. After removing long-term trends, net energy entering and exiting the climate system at the top of the atmosphere (TOA) should agree with the sum of energy entering and exiting the ocean, atmosphere, land, and ice over the course of an average year. Achieving such a balanced budget with observations has been challenging. Disagreements have been attributed previously to sparse observations in the high-latitude oceans. However, limiting the local vertical integration of new global ocean heat content estimates to the depth to which seasonal heat energy is stored, rather than integrating to 2000 m everywhere as done previously, allows closure of the global seasonal energy budget within statistical uncertainties. The seasonal cycle of energy storage is largest in the ocean, peaking in April because ocean area is largest in the Southern Hemisphere and the ocean’s thermal inertia causes a lag with respect to the austral summer solstice. Seasonal cycles in energy storage in the atmosphere and land are smaller, but peak in July and September, respectively, because there is more land in the Northern Hemisphere, and the land has more thermal inertia than the atmosphere. Global seasonal energy storage by ice is small, so the atmosphere and land partially offset ocean energy storage in the global integral, with their sum matching time-integrated net global TOA energy fluxes over the seasonal cycle within uncertainties, and both peaking in April.

**Article Highlights**

- Earth’s global seasonal energy storage budget closes when ocean storage is limited to 50 m below the surface mixed layer maximum depth
- Global ocean seasonal heat storage is maximum in April, consistent with its larger Southern Hemisphere area and massive thermal inertia
- Global atmospheric seasonal energy storage is maximum in June, land in September, consistent with larger Northern Hemisphere land area

## Introduction

In recent decades, the ocean, atmosphere, and land have all been warming substantially and ice (at sea and on land) has been melting, primarily owing to the buildup of greenhouse gases in the atmosphere from human activities. The amounts of energy required for these changes have been estimated globally (e.g., von Schuckmann et al. 2020). To estimate the time history of heat energy storage in the ocean, measurements of ocean temperature have been mapped on their own over time or assimilated in ocean models, with the results of the latter being known as reanalyses. Energy storage in the atmosphere is dominated by changes in temperature and moisture content (the latter necessitating phase changes which require substantial energy). In the atmosphere measurements assimilated into models producing reanalyses are the primary means for assessing the time history of atmospheric energy storage. Over long time scales, land warming has been estimated mostly from borehole temperature profiles (Beltrami et al. 2002), but for shorter time scales, land process models of varying complexity are driven with atmospheric reanalysis fields to estimate the time history of changes in land heat energy storage (e.g., Fasullo and Trenberth 2008; McKinnon and Huybers 2016). Sea ice areas are measured by satellites (e.g., Parkinson and Cavalieri 2008), and sea ice volumes in recent years may be estimated using satellite observations of sea ice thickness (e.g., Kwok and Rothrock 2009; Laxon et al. 2013). Sea ice volumes in all seasons may be estimated also using reanalysis sea ice models that are often constrained by satellite observations of sea ice concentration via data assimilation (e.g., Zhang 2014; Zhang and Rothrock 2003). Land ice mass changes can be derived from satellite gravimetry observations (e.g., Velicogna 2009).

On decadal and longer time scales, ocean heat storage dominates increases in energy storage in the climate system, accounting for about 90% of the total energy taken up in recent decades according to observational estimates (von Schuckmann et al. 2020). However, observational estimates of ocean warming are noisy to varying extents (Trenberth et al. 2016) even in recent years, with month-to-month ocean heat content anomaly (OHCA) estimates using Argo data alone or all available in situ data having about three times the variance of top of the atmosphere (TOA) radiation budgets from satellite measurements (Lyman and Johnson 2023). Even on annual time scales, variations in the rates of 0–2000 m ocean heat storage rates generally have larger variances than TOA net energy fluxes and the two independent measurements are at best correlated at less than 0.8 (e.g., Johnson et al. 2016). However, ocean warming estimates and TOA energy flux measurements both show a substantial and statistically significant increase in energy uptake by Earth’s climate system in recent years, with the rate roughly doubling from 2005 to 2019 according to these independent measurement systems (Loeb et al. 2021).

Over six months, the change of globally integrated seasonal cycles of energy storage may exceed 60 ZJ (e.g., Pan et al. 2023). Hence, energy moves in and out of the global ocean on seasonal time scales at an order of magnitude larger than the average rate of long-term uptake of energy by the global ocean over the past few decades, which is on the order of 10 ZJ yr^−1^ (e.g., Loeb et al. 2021). The percentage contributions to the seasonal cycle of globally averaged energy storage in the climate system from the atmosphere and land are larger than their percentage contributions to the long-term energy increase, but the ocean still dominates, with a seasonal cycle that is larger than that of the net flux at the TOA as shown by Fasullo and Trenberth (2008) and McKinnon and Huybers (2016). However, in both of these previous studies, the sums of the individual components’ (ocean’s, atmosphere’s, land’s, and ice’s) global seasonal cycles of energy storage do not agree within uncertainties with the net TOA energy storage. That is to say, the seasonal cycles of energy storage do not close. In both of those studies, this disagreement was attributed to poorly quantified measured or completely absent estimates of ocean heat storage in sparsely measured regions (e.g., the marginal seas and the Arctic Ocean) in the OHCA maps analyzed.

Here, we revisit the seasonal cycle of energy storage in light of a longer time series of Argo data and new high resolution (1/4° × 1/4° × 7 day) maps of OHCA that use Argo and other in situ OHCA data to train a random forest regression with satellite sea surface height, sea surface temperature, location, and time as predictors (Lyman and Johnson 2023). These maps include estimates of OHCA in the Arctic and most marginal seas (with exceptions being the Black and Caspian seas). Their global integrals are also substantially less noisy than maps made with in situ temperature profiles only, and their time derivative is generally better correlated with global integrals of CERES TOA net energy fluxes on time scales from monthly to annual, with or without the seasonal cycle included (Lyman and Johnson 2023). Nonetheless, with these maps, despite their coverage of high latitudes and most marginal seas, here we find lack of closure very similar to previous studies when using 0–2000 m depth integrals of OHCA. However, we can close Earth’s seasonal global energy budget when integrating from the surface to 50 m deeper than the maximum local monthly 95th percentile mixed layer depth taken from Johnson and Lyman (2022). That depth range encompasses the portion of the ocean where energy is likely to be exchanged with the atmosphere on seasonal time scales.

## Data and Methods

Weekly OHCA values constructed using Argo and other in situ ocean temperature data to train random forest regressions using location, time, satellite sea surface height anomaly maps, and satellite sea surface temperature maps (Lyman and Johnson 2023) are locally depth integrated from the surface to 2000 m and then globally integrated for comparison with previous studies. They are also locally depth integrated from the surface to 50 m deeper than the maximum monthly climatological 95th percentile mixed layer depth from Johnson and Lyman (2022) and then globally integrated. Where that mixed layer depth is not available (mostly in ice covered high latitudes), the integration is from the surface to 130 m, which should be sufficiently deep to encompass the ocean diabatic seasonal cycle in heat storage in those regions. In both cases, the resulting OHCA values are smoothed with a one-month half-power loess filter and interpolated to mid-months. The results presented below are very similar if the interpolation is performed on the unsmoothed time series, or if the smoothing varies between 0.5 and 1.5 months. Once the interpolation is increased to > 1.5 months, the fidelity of the seasonal cycle of OHCA begins to be compromised sufficiently that the closure achieved below is reduced.

Monthly means of total atmospheric energy are obtained using output from the fifth generation European Re-Analysis (ERA5; Hersbach et al. 2020). ERA5 combines a wealth of atmospheric observations (remotely sensed and in situ) with a dynamical model using four-dimensional variational data assimilation to provide physically consistent fields of atmospheric state and flux quantities with ~ 30 km spatial resolution on 137 vertical levels. The ERA5 archive provides monthly means of all output fields, which facilitates computation of monthly mean atmospheric energy content. Atmospheric total energy encompasses internal and potential energy (which can be combined to give sensible heat), latent heat, and kinetic energy, the latter being small (see, e.g., Peixoto and Oort 1992). In this study, we compute total energy on the native horizontal grid, integrate vertically using model level data, and from this compute global averages. Uncertainties of atmospheric state quantities like temperature and moisture from reanalyses are deemed relatively small since they are well constrained by a large number of observations, especially in the study period of the present work. Even in the Arctic, where observations are relatively sparse, the systematic differences in the seasonal cycle of atmospheric energy storage from three state-of-the-art reanalyses are fairly small, much smaller compared to uncertainties in energy storage in other components of the system (Mayer et al. 2019).

Monthly land energy flux estimates are obtained from the Community Land Model version 5 (Lawrence et al. 2019; https://doi-org.cuucar.idm.oclc.org/10.1029/2018MS001583), run to produce a historical reanalysis following the TRENDY Protocol (Friedlingstein et al. 2020), forced with CRU TS version 4.03 fields (Harris and Jones 2020; Harris et al. 2020) prior to 1954 and JRA55 fields (Kobayashi et al. 2015) from 1955 through 2020, both at 0.5° spatial resolution. Greenland and Antarctica are omitted from the area integration, as those regions are largely covered by ice sheets, and the energy required to melt those ice sheets is estimated from the time variation of satellite gravity measurements as detailed below. The global integrals are integrated in time to obtain a cumulative land energy storage estimate and then interpolated to mid-months.

Monthly global sea ice volumes (Arctic and Antarctic) are estimated using a coupled global sea ice–ocean model, the Global Ice–Ocean Modeling and Assimilation System (GIOMAS) (Zhang 2014; Zhang and Rothrock 2003). The ocean model component of GIOMAS is from the Parallel Ocean Program developed at the Los Alamos National Laboratory (Smith et al. 1992). The sea ice model component employs the multicategory ice thickness and enthalpy distribution model (Zhang and Rothrock 2003). It has 8 sub-grid categories each for ice thickness, ice enthalpy, and snow depth, representing variable ice thicknesses up to 28 m. GIOMAS assimilates satellite observations of sea ice concentration. Sea ice volume changes are converted to energy storage changes assuming an average density of 910 kg m^−3^ and a heat of fusion of 3.3 × 10^5^ J kg^−1^ for sea ice.

Monthly land ice mass variations are estimated from the Gravity Recovery and Climate Experiment (GRACE) and GRACE follow-on satellite data records (Landerer et al. 2020) using the JPL (Jet Propulsion Laboratory) Mascon data (Watkins et al. 2015; Wiese et al. 2019). Here, we sum ice mass changes over Greenland and its peripheral glaciers and Antarctica. The ice mass variations are then converted to energy changes following Slater et al. (2021, their Eq. 1 with Δ*T* = 10 °C).

Globally integrated TOA monthly averages of net energy flux from the Clouds and the Earth’s Radiant Energy System (CERES) Energy Balanced and Filled (EBAF) Edition 4.1 product (Loeb et al. 2018) are integrated in time to convert them from energy flux into energy storage values and then interpolated to mid-months. The CERES EBAF product provides monthly mean TOA shortwave (SW), longwave (LW), and net radiative fluxes on a 1° × 1° grid from March 2000 onward. Net radiative fluxes are calculated from the difference between spatially and temporally averaged monthly solar irradiances and the sum of outgoing SW and LW fluxes. The EBAF product uses an objective constraint algorithm (Loeb et al. 2009) to adjust SW and LW TOA fluxes within their ranges of uncertainty to remove the inconsistency between the 10-year average (07/2005–06/2015) global net TOA energy flux and energy storage in the earth–atmosphere–land–ice climate system, determined primarily from OHCA data (Johnson et al. 2016). Use of this approach to anchor the satellite-derived Earth energy imbalance to the in situ value does not affect the temporal variability in the data.

A quadratic function of time and six harmonics (with annual, semiannual, triannual, quarterly, five per year, and bimonthly periods) are fit to each time series starting in January 2008 and ending in December 2020. The harmonics are then used to re-construct the seasonal cycle. The quadratic and annual harmonics are also removed from the time series to create a residual time series. The variances of the residuals for each calendar month, together with the number of degrees of freedom, are used to construct 5–95% confidence limits for each of the monthly means. Hence, the confidence limits include only information about the variances from the average seasonal cycle for the 13 years analyzed, and neither any systematic nor possibly some random sources of error in the datasets. Therefore, they should be considered as a lower bound for the actual confidence limits. When summing or differencing estimates, the confidence limits are assumed to be independent. Hence, they are added in quadrature (taking the square root of the sum of the squares). Again, this assumption makes those sums and differences a lower bound on the actual confidence limits.

## Results

**Fig. 1:**
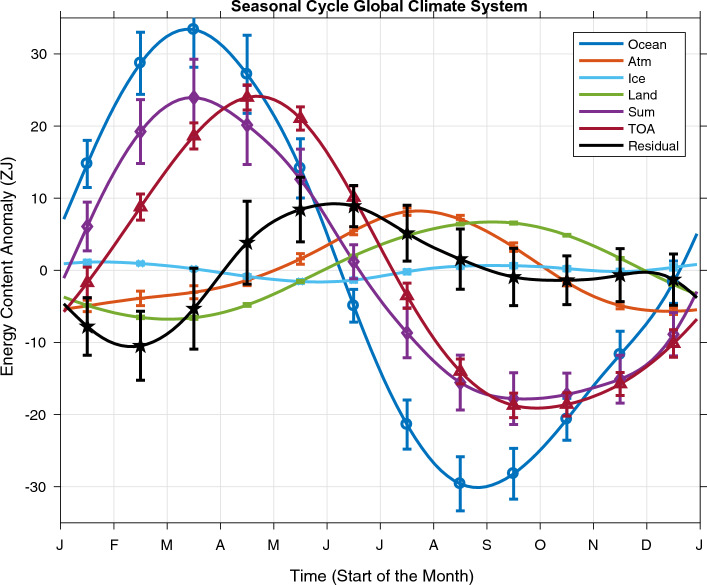
Seasonal cycles of energy storage for reservoirs in Earth’s climate system estimated from monthly values from January 2008 through December 2020 as described in the text. Ocean heat storage is estimated by integrating from the surface to 2000 m. The residual is the sum of energy storage in the various reservoirs minus that at the top of the atmosphere  (TOA). Error bars show 5–95% confidence intervals

We discuss the seasonal cycles in energy storage in the various components of Earth’s climate system roughly in order of amplitude from the largest to the smallest. We then compute their sum and compare it to the seasonal cycle of time-integrated net TOA energy flux, to assess whether or not the budget is closed. First, we present the seasonal cycle when integrating OHCA to 2000 m (Fig. 1), as previous studies (Fasullo and Trenberth 2008; McKinnon and Huybers 2016) have done, although the budget did not close in those previous studies, and does not close here.

The ocean integrated from 0 to 2000 m has the largest seasonal cycle of heat storage of the energy reservoirs in Earths’ climate system (Fig. 1, dark blue line with circles). In the global average, the ocean absorbs 63.5 ZJ of heat energy from late August (near the start of austral spring) to mid-March, a time period of around 7 months. It discharges that energy more rapidly, over a period of about 5 months. The standard deviation of the seasonal cycle of ocean heat storage is 22.1 ZJ.

The atmosphere (Fig. 1, orange line with pluses) and land (Fig. 1, green line) have comparably sized seasonal cycles of energy storage. The atmosphere takes up 13.9 ZJ of energy from mid-December through late July, a period of 7 months. Like the ocean, it discharges that energy over a period of 5 months. The standard deviation of the seasonal cycle of atmospheric energy storage is just under 4.8 ZJ. The land takes up 13.5 ZJ of heat from late February to early September, a period 6 months, and discharges that heat over an approximately equal period of time. The standard deviation of the seasonal cycle of land heat storage is just over 4.8 ZJ.

The cryosphere (Fig. 1, light blue line with crosses) accounts for the smallest portion of the seasonal energy storage, gaining 2.8 ZJ of energy in total from late May through late January, a period of 8 months, and losing it in four months. The standard deviation of the seasonal cycle of energy storage in the cryosphere is only 0.8 ZJ. However, the energy in the semiannual harmonic is more evident in ice energy storage than in the other components of Earth’s climate system, with a local maximum in energy storage in early September and a local minimum in mid-November.

Because of the opposing phasing of seasonal energy storage in the ocean versus the atmosphere and land reservoirs of Earth’s climate system, their sum (Fig. 1, purple line with diamonds, also including the smaller energy reservoirs of sea ice and ice sheets) has an amplitude smaller than that of the ocean. When integrating ocean heat storage from the surface to 2000 m, the seasonal cycle of the sum of energy storage in Earth’s climate system gains 41.8 ZJ of energy from late September through mid-March, a period of around 6 months, and discharges it again over 6 months. The standard deviation of the seasonal cycle of the sum of energy in Earth’s climate system is 15.3 ZJ.

The seasonal cycle of the integral of net energy flux through the TOA (Fig. 1, red line with triangles) is similar in amplitude to the seasonal cycle of the sum of energy storage in Earth’s climate system, but lags it by about a month. The seasonal cycle of the integral of net energy flux through the TOA gains 43.2 ZJ of energy from late September through late April, a period of about 7 months. Its standard deviation is 15.4 ZJ. Largely because of this mismatch in phase, the residual of the integral of the TOA energy flux minus the sum of storage in the ocean, atmosphere, land, and ice reservoirs (Fig. 1, black line with pentagrams) gains 9.8 ZJ of energy from mid-February though early July, with substantial energy in some of the higher harmonics evident, and a standard deviation of 5.8 ZJ, larger than that of either the land or the atmosphere. Furthermore, the residual is statistically significantly different from zero for five months of the year, so the global seasonal energy budget does not close within uncertainties.

**Fig. 2:**
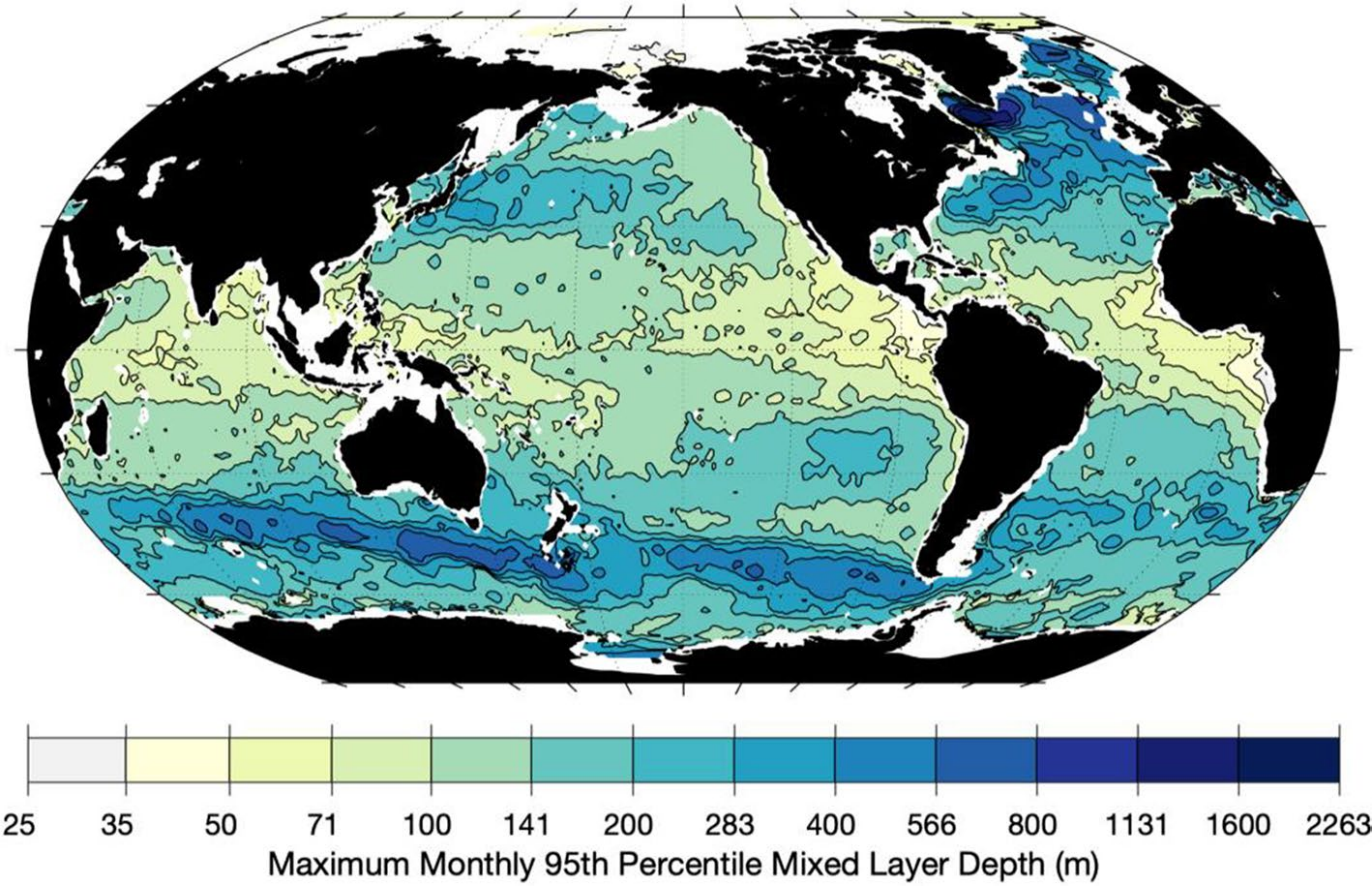
Maximum monthly climatological 95th percentile mixed layer depth from the Global Ocean Statistical Mixed Layer (GOSML) climatology (Johnson and Lyman 2022)

**Fig. 3:**
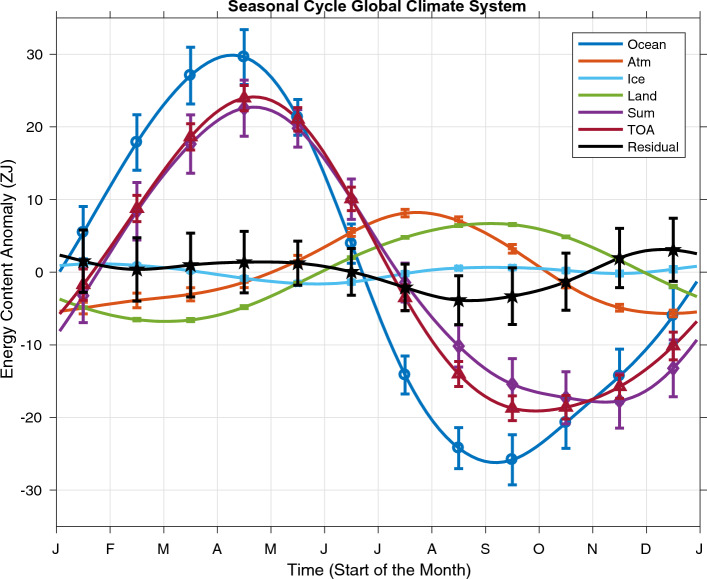
Seasonal cycles of energy storage for reservoirs in Earth’s climate system estimated from monthly values from January 2008 through December 2020 as described in the text. Ocean heat storage is estimated by integrating from the surface to 50 m below the maximum monthly 95th percentile mixed layer depth from Johnson and Lyman (2022), or from the surface to 130 m where those values are not mapped, primarily in ice-covered high-latitude regions where that depth of integration should encompass the seasonal cycle of ocean diabatic heat energy storage. The residual is the sum of energy storage in the various reservoirs minus that at the TOA. Error bars show 5–95% confidence intervals

Limiting the depth of integration of OHCA from the surface to just 50 m deeper than the maximum climatological 95th percentile monthly mixed layer depth (Fig. 2) from Johnson and Lyman (2022) changes this result and allows the global seasonal energy budget (Fig. 3) to close within uncertainties. In the global average, the ocean (Fig. 3, dark blue line with circles) absorbs 56.1 ZJ of heat energy from early October (near the start of austral spring) to mid-April, a time period of about 7 months. It discharges that energy more rapidly, over a period of about 5 months. The standard deviation of the seasonal cycle of ocean heat storage is 19.6 ZJ.

The reduction in amplitude and slight change in phase of ocean heat storage when limiting the integration from the surface to just 50 m deeper than the maximum climatological 95th percentile monthly mixed layer depth means that the seasonal cycle of the sum of the energy storage reservoirs (Fig. 3, purple line with diamonds) gains 40.6 ZJ of energy from mid-November through mid-April, with a standard deviation of 14.6 ZJ, pretty closely matching the seasonal cycle of the time-integrated global TOA net energy flux (Fig. 3, red line with triangles). The residual of the time-integrated global TOA net energy flux minus the sum of the global energy storage reservoirs (Fig. 3, black line with pentagrams) now gains just 7.0 ZJ of energy from mid-August to mid-December, with substantial variance in semi-annual and shorter period harmonics. The standard deviation of seasonal cycle of the residual is 2.1 ZJ, much less than that for any of the other terms in the budget except for the ice volume reservoir (Fig. 3, light blue line with crosses). Furthermore, only the monthly value of the residual for August is barely statistically significantly different from zero at 5–95% confidence limits. Given that there are 12 months in a year, this result is still consistent with a budget that is closed within uncertainty.

## Discussion and Conclusions

Previous analyses (Fasullo and Trenberth 2008; McKinnon and Huybers 2016) have noted that the seasonal cycle of the globally integrated energy storage by Earth’s climate system was not closed, and attributed that to either errors in estimates or absence of estimates of the ocean’s seasonal cycle in 0–2000 m temperature at high latitudes. Here, we attempt a similar exercise with near-global OHCA maps that extend well into the high latitudes, yet still get a similar result (Fig. 1) to those previous studies. As in those studies, the seasonal cycle in 0–2000 m ocean heat storage amplitude is too large and peaks too early in the year for seasonal energy budget closure, resulting in a monthly residual that has more variance over the year than the seasonal cycles of either land or atmospheric energy storage.

However, if we limit the local depth of OHCA integration to from the ocean surface to 50 m below the maximum monthly 95th percentile mixed layer depth (Fig. 2) from Johnson and Lyman (2022), the seasonal energy budget does close (Fig. 3), with the seasonal cycle of the sum of the globally integrated energy storage reservoirs (ocean, atmosphere, land, and ice) agreeing within uncertainties to the time-integrated TOA global net energy flux. This procedure is well justified, since diabatic seasonal heating is limited to slightly deeper than the deepest seasonal mixed layer (Moisan and Niiler 1998). Furthermore, seasonal adiabatic heave of isotherms is found in many locations below the seasonal thermocline. For example, prominent annual Rossby waves extend well below the permanent thermocline in the tropics of all three oceans (Brandt and Eden 2005; Johnson 2011; Kessler and McCreary 1993; Nagura 2018; Zanowski et al. 2019). We varied the depth added to the maximum monthly 95th percentile mixed layer depth from 0 to 100 m without an appreciable change in results (not shown). In fact, simply integrating in depth from the surface to 290 m gave a very similar result of budget closure (also not shown), so the finding seems fairly robust.

When OHCA is globally integrated from 50 m below the maximum depth of the seasonal mixed layer to 2000 m, this isotherm heave results in a substantial seasonal adiabatic signal (the difference between the seasonal cycles of OHCA in Figs. 2 and 3). This deep heave signature has a cooling effect in April–September and a warming effect in November–March. A recent global analysis of the seasonal cycle in ocean temperature using the mean of ocean temperature maps from seven different groups shows the global integral of this deep dynamically driven heave signal propagating down to at least 1000 m (Pan et al. 2023, their Fig. 8a), and our maps (not shown) confirm that this propagation extends to at least 2000 m.

The only way to eliminate this adiabatic heave signal would be to observe (and integrate over) the full ocean depth globally, as is proposed with the Deep Argo mission (Roemmich et al. 2019). Of course, for ocean heat gain over years to decades, integrating as deep as possible is desirable, since the ocean is gaining heat from the surface to the 2000 m sampling limit of core Argo floats (Wijffels et al. 2016), well below the deepest seasonal mixed layer, and even in the Antarctic Bottom Waters from 4000 to 6000 m (Desbruyeres et al. 2016; Purkey and Johnson 2010), where Deep Argo floats can sample.

The ocean, with its great mass, large heat capacity, and low albedo, dominates the seasonal cycle of energy storage within Earth’s climate system. Because the ocean’s surface area is much larger in the Southern Hemisphere than in the Northern Hemisphere, and it has substantial thermal inertia, its seasonal heat storage (Fig. 3, dark blue line with circles, integrated from the surface to 50 m below the depth of the maximum climatological 95th percentile monthly mixed layer depth) peaks in mid-April, well after the austral summer solstice in late December. Because of its low albedo and massive heat capacity, the ocean is the largest seasonal energy reservoir in Earth’s climate system, with a seasonal standard deviation of 19.6 ZJ.

The land (Fig. 3, green line) and atmosphere (Fig. 3, orange line with pluses) both have seasonal energy storage cycles with standard deviations of 4.8 ZJ, and so are reservoirs with very similar magnitudes in terms of Earth’s seasonal energy cycling. Hence, they each have ~ ¼ the standard deviation, or ~ 6% of the variance, of the ocean’s seasonal cycle of heat energy storage. Land area is much larger in the Northern Hemisphere than in the Southern Hemisphere, the atmosphere heats and cools much more seasonally over the land than over the ocean, and the atmosphere has relatively little thermal inertia. Hence, atmospheric energy storage peaks in mid-July, shortly after the boreal summer solstice. In contrast, seasonal energy storage by the land, with its considerably larger thermal inertia, peaks in mid-September, well after that of the atmosphere.

The seasonal cycle of energy storage in the cryosphere (Fig. 3, light blue line with crosses) has a standard deviation of 0.8 ZJ, making it by far the smallest of the seasonal energy reservoirs in Earth’s climate system. Hence it has ~ 4% the standard deviation, or ~ 0.2% of the variance, of the ocean’s seasonal cycle of heat energy storage. There is considerable semi-annual energy in the seasonal cycle, mostly because the rates of growth and decay of sea ice in both hemispheres are not symmetrical, although there is considerable cancellation of the hemispheric contributions in the global integral, as for the other reservoirs of energy.

The cooling periods for the globally integrated TOA, ocean, and atmosphere seasonal cycles of energy storage are all noticeably shorter, about five months, than their warming periods of about seven months. For the TOA, a likely contributing factor to this asymmetry is the planetary albedo: The globally averaged albedo (not shown) is higher than average during November through January, when highly reflective mid-latitude regions (clouds) and high latitude regions (Antarctic ice) in the Southern Hemisphere are illuminated, which induces asymmetry in the seasonal cycle of absorbed solar radiation. Additionally, TOA outgoing longwave radiation (not shown) has a relatively narrow maximum centered on July–August, with phasing and amplitude likely associated with Northern Hemisphere land heating, and a broader minimum centered on December. The Southern Hemisphere OHCA seasonal cycle (not shown) is highly symmetric, with ~ 99.99% of its variance in the annual harmonic, and has about 1.9 times the variance of the Northern Hemisphere OHCA seasonal cycle, primarily owing to the larger area of the ocean in the Southern Hemisphere. The Northern Hemisphere OHCA seasonal cycle has a cooling period of 5.6 months, probably owing to the greater fraction of land there. In boreal fall and winter, the atmosphere over land cools more quickly than that over the ocean, enhancing atmospheric land-to-ocean energy transport (e.g., Trenberth and Fasullo^ 2013^) and exchange of energy from the ocean to the atmosphere via turbulent fluxes (e.g., Cayan 1992)—thus likely accelerating, and shortening, the oceanic cooling period. The sum of the two largely opposing OHCA seasonal cycles in each hemisphere accentuates the asymmetry in the global OHCA seasonal cycle. For the atmosphere, one possible reason for the shorter cooling period could be Northern Hemisphere snow cover on land: Once there is a thin snow cover in boreal fall, ground heat flux is suppressed and atmospheric cooling accelerates, while snow cover in boreal spring decelerates warming, and hence the buildup of energy in the warming period takes longer.

The seasonal cycles of globally integrated energy storage in Earths’ climate system are substantial with respect to the rates of long-term energy accumulation in the reservoirs of that climate system. For instance, the global seasonal cycle of OHCA has rates of heat accumulation about an order of magnitude greater than the decadal increase in global OHCA. This attribute makes the seasonal cycle of global energy storage in the various reservoirs an important benchmark for climate models.

The sum of the seasonal cycle of energy storage in all the components of Earth’s climate system (Fig. 3, purple line with diamonds) overlaps with the time integral of net TOA energy flux (Fig. 3, red line with triangles) in all months within their 5–95% confidence limits, meaning that the budget closes (Fig. 3). The residual (Fig. 3, black line with pentagrams, sum minus TOA) has noticeable semi-annual and higher energy, but overall has a very small global seasonal cycle compared with all other seasonal climate system energy storage reservoirs except ice. The standard deviation of the seasonal cycle of the global residual is only 2.1 ZJ, considerably less than the mean 5–95% confidence interval of the residual, which is ± 3.4 ZJ. Furthermore, the variance of the seasonal cycle of the global residual is only 2% of the variance of the seasonal cycle of either the time-integrated net TOA energy flux or the seasonal cycle of the sum of energy storage in Earth’s climate system. Only one monthly mean, the minimum found in August, is barely statistically significantly different from zero at 5–95% confidence. This marginal discrepancy of one month out of 12 is consistent with a closed budget at 5–95% confidence limits. This result suggests that our choice of depth integrating ocean heat content from the surface to 50 m below the maximum 95% monthly climatology mixed layer depth is capturing the seasonal ocean heat storage, as expected.
